# Community Awareness About Developmental Dysplasia of the Hip (DDH) in the Western and Southern Regions of Saudi Arabia

**DOI:** 10.7759/cureus.58442

**Published:** 2024-04-17

**Authors:** Zenat A Khired, Basem Zogel, Hussam Darraj, Rana K Asiri, Yasser B Hennawi, Sultan M Alhazmi

**Affiliations:** 1 Department of Surgery, Jazan University, Jazan, SAU; 2 Department of Medicine and Surgery, Jazan University, Jazan, SAU; 3 Department of Surgery, College of Medicine, Jazan University, Jazan, SAU; 4 College of Medicine, Umm Al-Qura University, Makkah, SAU; 5 College of Medicine, Jazan University, Jazan, SAU

**Keywords:** saudi arabia, ddh, developmental dysplasia of the hip, awareness, knowledge

## Abstract

Background: Developmental dysplasia of the hip (DDH) is a disorder in which the hip joint does not develop normally in the pediatric age group. It is caused by a confluence of hereditary and environmental factors. We aimed to examine knowledge and awareness of DDH among the general population of the southern and western regions of Saudi Arabia.

Methodology: A cross-sectional survey-based study was conducted in the western and southern regions of Saudi Arabia. This study included adult male and female participants above 18 years of age. Data were collected using a validated electronic questionnaire that was disseminated via social media platforms. All data were analyzed using IBM SPSS Statistics for Windows, Version 23.0 (IBM Corp., Armonk, NY).

Results: In this study, 1,232 participants were surveyed in Saudi Arabia. The majority were between 21 and 30 years old (663, 53.8%), unmarried (690, 56%), and had a baccalaureate or diploma certificate (886, 71.9%). Regarding knowledge of DDH, 86.4% of participants had poor knowledge of the causes of DDH, and 740 (60%) had poor overall knowledge of DDH. However, 492 (40%) participants had good knowledge. Respondents with a higher monthly income, those who were mothers, and those who obtained information from social media had a better awareness level. Concerning treatment, 531 (43.1%) participants were unsure about the best treatment for DDH, and 850 (69%) believed that early treatment was better.

Conclusions: According to our literature, DDH is highly prevalent among Saudi populations. However, our findings indicate that the majority of the Saudi population residing in the western and southern regions of Saudi Arabia lacks basic knowledge of DDH. All capable facilities, such as medical schools, hospitals, and primary healthcare centers, must impart cultural education about DDH to address this awareness gap.

## Introduction

Developmental dysplasia of the hip (DDH) is a spectrum of hip disorders in which the hip's *ball and socket* joint fails to develop correctly in newborns and young children. The hip socket is too shallow in DDH, and the femoral head is not kept snugly in place, resulting in a loose hip joint. The femur head may come out of the socket in extreme situations (dislocate), acetabular dysplasia with or without subluxation and dislocation. The clinical examination with hip ultrasound remains the most significant part of the evaluation process at childbirth, within 72 hours, and between six and eight weeks of age at well-baby clinics [1]. Barlow and Ortolani's maneuvers are used in the examination. The hip is flexed and abducted during the Ortolani maneuver as the greater trochanter is gently pulled and pressed. The reduction of the dislocated hip indicates positive results. An urgent referral for treatment is required if the test for hip dislocation is positive.

Barlow maneuver

If the hip can be manually dislocated, it is usually adducted, and pressure is relieved from the greater trochanter. Consider getting an urgent ultrasound between four and six weeks with an equivocal examination for selective screening for newborns with risk factors or offering additional anatomical details for the diagnosis [2]. DDH is caused by a confluence of hereditary and environmental factors. The transforming growth factor beta superfamily, essential for normal bone and joint development, has associated gene abnormalities [2]. The left hip is the most commonly affected. It is also common among girls, firstborns in families with a history of hip difficulties in children (parents, brothers, or sisters), and infants delivered in the breech position (feet or bottom down) after 28 weeks of pregnancy. These are additional groups where the condition is more prevalent [1].

The DDH presentation varies with age, and the pathologic changes range from mild abnormalities to dislocations [1]. Therefore, DDH can be screened by clinical examination, and the diagnosis can be confirmed through radiological methods such as ultrasounds [3]. The treatment of DDH focuses on the positioned femoral head into the acetabulum [4]. This goal can be achieved in various ways, such as utilizing a hip abduction splint (Pavlik harness), primarily employed in children under four months with dislocated hips. Its usage should be supervised closely by a mentor, and reevaluation after two weeks is necessary to ensure the correct positioning of the femur. Closed and open reduction plus spica casting can also be used in case of hip abduction splint failure. If there is an anatomical abnormality in the proximal femoral, femoral osteotomy should be considered to achieve the reduction. Additionally, in late-presenting children, due to the unpredictable remodeling potential of the acetabulums, acetabular osteotomy can be done [5]. A systematic review conducted in Saudi Arabia shows a prevalence of DDH of about 10.46 per 1000 in the Saudi population, with the majority of cases presenting after 12 months [6]. A cross-sectional study was conducted in the Aseer region among 253 pregnant women. About 5% of the women said they had a child with DDH, and 65.6% of pregnant women knew about it. In addition, 43.5% of the women knew how DDH is managed, and 39.1% were informed about the consequences of DDH.

A better level of knowledge was observed among multiparous females who had a child with DDH and those who obtained their information through study or work in the medical sector [7]. Some studies assessed the awareness regarding DDH in community categories other than females. A cross-sectional study was conducted to determine the knowledge and attitudes of primary care physicians toward DDH and develop further education and training programs, according to the results obtained from the study. They found that only 27.5% of the physicians were aware of the wrong traditional attitude considered risk factors for DDH. They recommend providing continuous education to programmers [8] and yet articles discussing people's knowledge, awareness, and attitudes toward DDH diagnosis and risk factors are scarce. This study aims to assess the community awareness of DDH and the knowledge of its risk factors, treatment, and complications among the population in Riyadh, Saudi Arabia.

## Materials and methods

Study design and participants 

This was a cross-sectional observational study employing a self-administered questionnaire. This study was undertaken in the western and southern regions of Saudi Arabia, which have a population of 10.4 million people. We included all adult male and female participants living in the western and southern regions of Saudi Arabia. Participation was restricted to participants who were at least 18 years old and willing to participate and complete the anonymous survey. We excluded all males and females below 18 years of age who refused to participate and did not complete the anonymous survey.

Ethical consideration and sampling strategy

Self-administered survey through Google Forms was used to collect data. The survey was distributed to the Saudi community on social media platforms such as WhatsApp, Telegram, and Twitter. The ethical approval was obtained from the Jazan Ethics Committee at Jazan University, Kingdom of Saudi Arabia, with approval number IRB: REC-44/07/537. 

Using Raosoft sample size calculators based on the population of the western and southern regions of Saudi Arabia, the minimum sample size required to achieve a precision of 5% with a 95% confidence interval was 385 participants.

Study tool 

Our study included 1,232 participants selected using a convenience sampling technique using an online self‑reported Arabic questionnaire. Several social media platforms were used to publicize it to the Saudi community, including WhatsApp, Telegram, and Twitter, by using data collectors in each region. The questionnaire was referenced from orthopedic literature and revised by an orthopedic clinician. The questionnaire was conducted through a pilot test of 21 participants to explore if there were any remaining ambiguities in the questionnaire, followed by face validity by sending the survey to an orthopedic surgeon.

The questionnaire language was further modified according to the difficulties and notes of the participants until we arrived at the final questionnaire.

Our survey consisted of two sections. The first section dealt with the sociodemographic characteristics of the participants (age, education, marital status, occupation, and nationality) and questions to assess the knowledge about DDH. The second section explored the participants’ awareness of DDH and its risk factors and management options.

The questionnaire was received by 1,767. Many of the candidates (535) did not meet the criteria and were excluded.

Data analysis 

IBM SPSS Statistics for Windows, Version 23.0 (IBM Corp., Armonk, NY) was used to analyze the data. For all variables, descriptive analysis based on frequency and percentage distribution was performed. To evaluate individuals' attitudes, knowledge, and awareness of DDH, the chi-square test was used. The final score was calculated by adding the points awarded for each correct answer and all discrete elements. Participants were considered to have low awareness levels if their overall score was 4 points or less, whereas those with an overall score of 5 or more were considered to have high awareness. Statistical significance was considered to be significant when the two-sided *P*-value was less than 0.05. The results are displayed as tables and graphs. 

## Results

Baseline sociodemographic characteristics and personal risk factors

The study’s authors distributed an electronic survey. The total number of participants was 1,232, with the majority falling within the age range of 21 to 30 years, accounting for 53.8% (663 participants). This was followed by participants aged between 31 and 40 years, comprising 14.6% (180), and those aged between 41 and 50 years, accounting for 14.1% (174). In terms of region, the majority resided in the western region of the Kingdom of Saudi Arabia, accounting for 56.6% (697), while the remaining participants lived in the southern region of the Kingdom, constituting 43.4% (535).In terms of marital status, the majority of participants were unmarried, comprising 56% (690), followed by those who were married, accounting for 42% (517). Regarding educational level, the majority of participants held a baccalaureate or diploma certificate, constituting 71.9% (886), followed by those with a high school certificate, accounting for 19.7% (243). In terms of monthly income, the majority of participants had an income below 5,000, with a rate of 51.1% (630), followed by those whose salaries ranged between 10,000 and 20,000, accounting for 23.5% (290). The majority of participants, comprising 61.5% (758), did not have children. When inquiring about their relationship with children, it was found that the majority of participants were aunts or uncles of children, accounting for 46.7% (575) (Table 1).

**Table 1:** Biodemographic data of participants. Data are represented as *n* (%).

Variable	Frequency (proportion)
Age (in years)	
18-20	123 (10%)
21-30	663 (53.8%)
31-40	180 (14.6%)
41-50	174 (14.1%)
51-60	76 (6.2%)
>60	16 (1.3%)
Regions	
Southern region of the Kingdom	535 (43.4%)
Western region of the Kingdom	697 (56.6%)
Marital status	
Married	517 (42%)
Single	690 (56%)
Widow	4 (0.3%)
Divorced	21 (1.7%)
Educational level	
Illiterate	2 (0.2%)
Primary school	6 (0.5%)
Intermediate school	13 (1.1%)
High school	243 (19.7%)
Bachelor’s degree or diploma	886 (71.9%)
Postgraduate	82 (6.7%)
Monthly income	
<5,000	630 (51.1%)
5,000-10,000	221 (17.9%)
10,000-20,000	290 (23.5%)
20,000-30,000	58 (4.7%)
>30,000	33 (2.7%)
Do you have children?	
No	758 (61.5%)
Yes	474 (38.5%)
Your relationship with this child	
Father	267 (21.7%)
Mother	206 (16.7%)
Don’t have a child	184 (14.9%)
An aunt or uncle	575 (46.7%)

Based on the analyzed data, participants were asked about the influencing factors of DDH, and in response to the first question regarding the presence of a child with DDH, the majority (1,206, 97.9% participants) answered no, while 26 (2.1%) reported that their children had been affected. When asked about the timing of the diagnosis, the majority were diagnosed at birth up to six months, accounting for 1.4% (17), followed by six months to a year, with a rate of 0.4% (5). Additionally, an equal percentage of participants, 0.2% (2), reported being diagnosed within one or two years. When asked about their knowledge of the causes of DDH, the majority of participants answered *No*, comprising 63.9% (787). Those who answered *Yes* accounted for 36.1% (445). Out of the 445 participants who answered *Yes*, 306 (68.7%) responded *No* when asked about the genetic causes. When asked whether the sitting position of the mother during pregnancy is one of the reasons for DDH, the majority of participants answered *Yes*, accounting for 50.3% (224). Regarding the method of childbirth as a cause of DDH, the majority of participants answered *Yes *with a percentage of 85.6% (381), while those who answered *No* comprised 14.3% (64). The majority of participants, comprising 48.9% (603), did not know whether people with DDH could walk or not, while 36.4% (449) believed they could, followed by 14.6% (180) who answered no. When asked about the preventability of DDH, most participants, accounting for 45.7% (563), indicated they did not know. This was closely followed by 45.6% (562) who answered yes, and finally, 8.7% (107) who answered no. When asked whether a low quantity of amniotic fluid during pregnancy could lead to developmental dysplasia in the hip, the majority of participants, comprising 55.8% (688), indicated they did not know. Regarding their belief about the relationship between the method of childbirth and DDH, the majority answered yes, accounting for 68.1% (839), followed by 28% (345) who were unsure (Table 2).

**Table 2:** Awareness levels regarding DDH joint in the general population in the western and southern regions of Saudi Arabia. Data represented as *n* (%). DDH, developmental dysplasia of the hip

Factor	Frequency	Percentage
Do you have children with hip displacement?		
Yes	26	2.1
No	1,206	97.9
How old was your child at the time of diagnosis?		
From birth to six months	17	1.4
Six months to one year	5	0.4
One year	2	0.2
Two years	2	0.2
Don't have a diagnosed child	1,206	97.8
Know the causes of DDH		
Yes	445	36.1
No	787	63.9
If participants answer (yes, *n *= 445) to the above question have to choose causes		
Genetic causes		
Yes	139	31.2
No	306	68.7
Setting position during pregnancy		
Yes	224	50.3
No	221	49.6
Mode of delivery		
Yes	381	85.6
No	64	14.3
Child gender		
Yes	74	16.6
No	371	83.3
Do you think that a child with DDH could walk?		
Yes	449	36.4
No	180	14.6
Don’t know	603	48.9
Do you think the disease is preventable?		
Yes	562	45.6
No	107	8.7
Don’t know	563	45.7
Hip dislocation is painful for the child		
Yes	746	60.6
No	91	7.4
Don’t know	395	32.1
During pregnancy, if the fluid around the fetus is low in amount, developmental dysplasia of the hip can develop.		
Yes	337	27.4
No	207	16.8
Don’t know	688	55.8
Do you think that mode of delivery is related to DDH?		
Yes	839	68.1
No	48	3.9
Don’t know	345	28

Table 3 presents the results of a study examining the relationship between sociodemographic data and the awareness level of respondents. As shown in Table 3, the majority of the participants (740, 60% participants) had poor knowledge regarding DDH, whereas approximately 492 (40%) participants had good knowledge. However, respondents with a higher monthly income, mothers, and those who obtained information from social media had a better awareness level than others (*P *= 0.047, 0.046, and 0.0001, respectively). Additionally, respondents who had children with displacement had a significantly higher awareness level (*P*-value = 0.0001). However, no significant relationship was found between age, region, marital status, education level, and the awareness level of the respondents.

**Table 3:** Distribution of awareness level among participants regarding DDH by their biodemographic data. *P*-values in bold indicate statistical significance. *P*-value < 0.05 is considered significant. DDH, developmental dysplasia of the hip

Factor	Awareness level				*P*-value**
	Poor (*n *= 740)		Good (*n *= 492)		
	n	%	n	%	
Age (in years)					0.097
18-20	86	69.9%	37	30.1%	
21-30	381	57.5%	282	42.5%	
31-40	115	63.9%	65	36.1%	
41-50	107	61.5%	67	38.5%	
51-60	41	53.9%	35	46.1%	
>60	10	62.5%	6	37.5%	
Regions					0.967
Southern region of the Kingdom	321	60%	214	40%	
Western region of the Kingdom	419	60.1%	278	39.9%	
Marital status					0.255
Married	318	61.5%	199	38.5%	
single	406	58.8%	284	41.2%	
Widow	1	25%	3	75%	
Divorced	15	71.4%	6	28.6%	
Education level					0.063
Illiterate	0	0.0%	2	100%	
Primary school	5	83.3%	1	16.7%	
Intermediate school	10	76.9%	3	23.1%	
High school	160	65.8%	83	34.2%	
Bachelor’s degree or diploma	518	58.5%	368	41.5%	
Postgraduate	47	57.3%	35	42.7%	
Monthly income					0.047*
<5,000	382	60.6%	248	39.4%	
5,000-10,000	139	62.9%	82	37.1%	
10,000-20,000	176	60.7%	114	39.3%	
20,000-30,000	31	53.4%	27	46.6%	
>30,000	12	36.4%	21	36.6%	
Do you have children?					0.933
No	456	60.2%	302	39.8%	
Yes	284	59.9%	190	40.1%	
Your relationship with this child					0.046*
Father	174	65.2%	93	34.8%	
Mother	110	53.4%	96	46.6%	
Don’t have a child	104	56.5%	80	43.5%	
An aunt or uncle	352	61.2%	223	38.8%	
Do you have children with displacement?					0.0001*
No	735	60.9%	471	39.1%	
Yes	5	19.2%	21	80.8%	
Source of information					0.0001*
Social media	76	45.5%	91	54.5%	
Friends and family	42	44.2%	53	55.8%	
College	0	0.0%	7	100%	
Self-education	72	26%	205	74%	
Doctors	20	23%	67	77%	

Table 4 shows the perception of participants about the treatment of DDH. Regarding the best treatment for DDH, 531 (43.1%) participants were unaware, while 283 (23%) believed that initially, no surgery was the best treatment. Two hundred eighty-one participants (22.8%) believed that surgery should be pursued only if the condition worsens, while 137 (11.1%) believed that surgical treatment was the best initial option. Regarding the prognosis after treatment, 524 participants (42.5%) were unaware, while 461 (37.4%) believed DDH could be completely healed. Two hundred thirty-four (19.0%) participants believed that DDH could be partially healed, but limping may persist, and only 13 (1.1%) believed that no treatment was required. Concerning early treatment, 850 (69%) participants believed that early treatment was better, while 350 (28.4%) were unaware. Only 20 (1.6%) believed that there was no difference between early and late treatment, and 12 (1%) believed that late treatment was better.

**Table 4:** Perception of participants about DDH treatment. Data represented as *n* (%). DDH, developmental dysplasia of the hip

Factor	Frequency	Percentage
What do you think is the best treatment for DDH?		
Initially surgical	137	11.1%
Initially no surgery	283	23%
Don't know	531	43.1%
Surgery if it gets worse	281	22.8%
What do you think is the prognosis after the treatment?		
No treatment	13	1.1%
Completely healed	461	37.4%
Don't know	524	42.5%
Partial healing with limping	234	19%
What do you think about early treatment?		
No difference	20	1.6%
Better	850	69%
Don't know	350	28.4%
Late treatment better	12	1%

## Discussion

DDH progresses over time and develops before and even after the birth of the child [9]. In Saudi Arabia, there is a very low level of awareness and knowledge about DDH, and different studies have been conducted to assess community awareness regarding DDH. In this study, a validated electronic questionnaire was distributed among participants through social media platforms to assess community knowledge and awareness of DDH in the southern and western regions of Saudi Arabia. It is important to note that all participants in this study had the independence to choose the correct answer whatever they believed as conducted in a previous study in Saudi Arabia [10]. Although DDH is preventable, it can increase the risk of life-threatening complications if not diagnosed early. Despite screening programs at the national level, a delayed diagnosis of DDH is still a hurdle in reducing the occurrence of the condition [11,12].

In our study, about 60% of participants had no idea about what DDH is, while 40% of the participants had a previous understanding of DDH, either from their child or a first-degree relative as the majority of participants in this study were aunts and uncles. This factor showed a correlation between our study and a previous study, which reported that respondents became aware of DDH because a family member was affected by it [10]. In comparison to another study conducted in 2022 in the Riyadh region, Saudi Arabia, which showed that 72.8% had a low knowledge level regarding DDH, our study's findings are consistent [13]. Around 48.9% had moderate awareness, and 37.8% had low awareness. In a more recent study conducted in Jordan in 2023, the results are almost close to our results [14]. 

The majority of the participants who knew DDH were mothers. This is not a surprising fact because mothers have long hours of contact with their infants and tend to be extra careful about their health. Since the objective of our study was to assess the level of community awareness about DDH, it was important to include the question "Do you have children with hip displacement?" at the beginning of the questionnaire. The study involved three steps: filtering the participants into two groups based on their prior knowledge of DDH, analyzing survey answers of these groups, and validating the accuracy of respondents’ answers. Consequently, we found a substantial relationship between previous knowledge of DDH, the source of information, whether a child with DDH was able to walk, and the preferred treatment for DDH. All these anticipations created a solid base for us to interpret our collected data. Our study also examined the awareness level of DDH by the source of information. Among the different sources of information, social media was the main source among a majority of participants, followed by friends and family, self-education, and doctors. This gives us the idea that awareness and knowledge of DDH can widely be discussed through social media platforms. A high awareness level (*P *= 0.0001) was also observed among participants who had children with DDH. Regarding employment, higher awareness rates of DDH were associated with higher education levels. In our study, participants holding a bachelor’s degree reported that DDH can be cured and preventable. In our study, we performed a comparison between low- and high-income participants to assess the level of awareness about DDH. The comparison revealed that participants with low income reported that their source of DDH knowledge was an affected family member, while high-income respondents reported that their source of DDH knowledge was self-education material. This comparison helped us construe how participants from low- and high-income classes showed their awareness level concerning their source of information. This can be helpful when awareness campaigns are launched to target people from different economic classes. It is because people from higher classes acquire DDH knowledge through different media channels, while people from lower classes do not put any effort into searching for information. It has been reported in the literature that the risk of DDH development in infants is higher when more than one risk factors are involved. This study also revealed that there is a twofold higher risk of having DDH in infants with at least one risk factor, while infants are more susceptible to having DDH when multiple risk factors are involved [15,16].

Having a family history of DDH is one of the major risk factors contributing to the development of DDH [17], and yet, the majority of participants (86.4%) in our study were not familiar with family history being the risk factor for DDH. Similarly, one study conducted by Alqarni et al. [7] reported that only a minor female population in Saudi Arabia knew the primary risk factors of DDH. Moreover, about 78.4% of our participants reported that they were unaware that the sitting position of the mother during their gestational period contributes to the higher risk of developing DDH. Yet again, the participants of our study had no idea that the method of childbirth also increases the risk for DDH. This showed that there was poor knowledge and awareness of DDH risk factors in our sample [18]. 

In our study, participants were also asked about the most suitable treatment. There was a high percentage of misconception among participants regarding the best treatment for DDH. Unfortunately, 23% of participants believed that surgery is not the best treatment for DDH. At the same time, 22.8% of participants preferred opting the surgical treatment only when the condition worsens. About half of the participants (43.1%) of our study were unsure whether surgery is the best treatment option for DDH. To expect the best possible outcomes and prevent extensive surgeries and long-term complications, early diagnosis and treatment interventions are critical [1]. In general, the overall community awareness in our study was found to be very low. Only 40% of participants had good knowledge about DDH, while the remaining 60% showed a lower level of awareness. A Saudi study involving participants in Riyadh reported that the results about the awareness of DDH were suboptimal. Furthermore, the awareness level, in this study, was much lower among fathers than mothers [13].

Since community awareness about DDH is suboptimal in Saudi Arabia, raising awareness by effectively educating community members can help decrease the incidence of DDH in Saudi Arabia. Establishing awareness campaigns through healthcare providers about complications of DDH and early intervention can increase knowledge among the Saudi population. Further, our study had some limitations.

Strengths and limitations

Our study was limited to only two geographical locations (southern and western regions of Saudi Arabia). Furthermore, our study utilized an electronic questionnaire that helped our researchers collect responses from southern and western regions of Saudi Arabia. As the questionnaire was self-administered, self-reporting bias might be present. There were very few studies investigating community awareness about DDH in Saudi Arabia. The findings of our study also highlighted the importance of healthcare professionals taking necessary steps (early diagnosis and treatment) to avoid long-standing complications of DDH [19]. We also encourage researchers and scholars to conduct more studies on DDH to further increase and examine awareness levels.

## Conclusions

DDH is highly prevalent among the Saudi population. However, our findings indicate that most of the Saudi population from the western and southern regions of Saudi Arabia are unaware of basic knowledge of DDH. Therefore, we suggest further extensive educational campaigns to increase awareness of the population to decrease the incidence of the complications related to it and improve the life healthcare of affected children.
